# Characteristics of Type-2 Diabetics Who are Prone to High-Cost Medical Care Expenses by Bayesian Network

**DOI:** 10.3390/ijerph17155271

**Published:** 2020-07-22

**Authors:** Yuji Sase, Daiki Kumagai, Teppei Suzuki, Hiroko Yamashina, Yuji Tani, Kensuke Fujiwara, Takumi Tanikawa, Hisashi Enomoto, Takeshi Aoyama, Wataru Nagai, Katsuhiko Ogasawara

**Affiliations:** 1 Faculty of Medical Informatics, Hokkaido Information University, Hokkaido 069-8585, Japan; sase@do-johodai.ac.jp; 2 School of Health Sciences, Hokkaido University, Hokkaido 060-0812, Japan; kd0703againxagain@gmail.com; 3 Art & Sports Business, Iwamizawa, Hokkaido University of Education, Hokkaido 068-8642, Japan; suzuki.teppei@i.hokkyodai.ac.jp; 4 Faculty of Health Sciences, Hokkaido University, Hokkaido 060-0812, Japan; yamashina-rt@hs.hokudai.ac.jp; 5 Department of Medical Informatics and Hospital Management, Asahikawa Medical University, Hokkaido 078-8510, Japan; y_tani@asahikawa-med.ac.jp; 6 Graduate School of Commerce, Otaru University of Commerce, Hokkaido 047-8501, Japan; k-fujiwara@res.otaru-uc.ac.jp; 7 Faculty of Health Sciences, Hokkaido University of Science, Hokkaido 006-8585, Japan; tanikawa-t@hus.ac.jp; 8 Iwamizawa City, Hokkaido 068-0828, Japan; h-eno@i-hamanasu.jp (H.E.); t-aoyama@i-hamanasu.jp (T.A.); w-nagai@i-hamanasu.jp (W.N.)

**Keywords:** health economics, bayesian network, diabetes, National Health Insurance, medical costs, specific health checkups

## Abstract

*Objective*: This study aims to determine the characteristics of Type 2 diabetic patients who are more likely to cause high-cost medical expenses using the Bayesian network model. *Methods*: The 2011–2015 receipt data of Iwamizawa city, Japan were collected from the National Health Insurance Database. From the record, we identified patients with Type 2 diabetes with the following items: age, gender, area, number of days provided medical services, number of diseases, number of medical examinations, annual healthcare expenditures, and the presence or absence of hospitalization. The Bayesian network model was applied to identify the characteristics of the patients, and four observed values were changed using a model for patients who paid at least 3607 USD a year for medical expenses. The changes in the conditional probability of the annual healthcare expenditures and changes in the percentage of patients with high-cost medical expenses were analyzed. *Results*: After changing the observed value, the percentage of patients with high-cost medical expense reimbursement increased when the following four conditions were applied: the patient “has ever been hospitalized”, “had been provided medical services at least 18 days a year”, “had at least 14 diseases listed on medical insurance receipts”, and “has not had specific health checkups in five years”. *Conclusions*: To prevent an excessive rise in healthcare expenditures in Type 2 diabetic patients, measures against complications and promoting encouragement for them to undergo specific health checkups are considered as effective.

## 1. Introduction

In 2015, the estimated number of diabetic patients in the world was 415 million (aged 20 to 79 years) with healthcare expenditures for diabetes amounting to 673 billion US dollars (converted at the exchange rate of 20 February 2019). In 2040, it is expected that the number of diabetic patients may increase to 642 million, and healthcare expenditures may increase to 802 billion US dollars [1]. Other studies have also reported that diabetes is a major financial burden in medical systems of the world. Therefore, we need to develop an approach that will reduce a large amount of the healthcare expenditure [2,3].

In 2016, the national healthcare expenditures in Japan accounted for 42,138,100 million JPY (379,756 million USD) and the per capita healthcare expenditures were 332,000 JPY (2994 USD). Changes in both national and per capita healthcare expenditures over the last 20 years show a tendency towards an increase [4]. Expenditure on health per person, including nursing care costs in Japan was 4150 USD, ranking 15th out of 35 Organisation for Economic Cooperation and Development (OECD) member countries (median was 3787 USD) [5]. In addition, per capita healthcare expenditures by age were 183,900 JPY (1658 USD) for people under 65, and 727,300 JPY (6559 USD) for people aged 65 years or older. In Japan, the number of elderly people have increased significantly; according to a survey conducted in 2017, the population 65 years or older was 35,140,000 and accounted for 27.7% of the total population. These figures are the highest they have ever been and are expected to increase even further, meaning a continued rise in national healthcare expenditures in the future. According to prefectural and city governments in Japan, Hokkaido has the sixth highest national medical expenses (2094 billion JPY (1.89 million USD)) and the eighth highest per capita healthcare expenditures (391,300 JPY (3529 USD)). Hokkaido is characterized by a decentralized population over a large area and extreme natural phenomena, such as snow coverage and cold conditions. There is a low number of people per household and a high percentage of households consisting of only elderly people.

According to a survey regarding the number of diabetic patients in Japan, both the number of “people with strongly suspected cases of diabetes” and the number of “people for whom diabetes cannot be ruled out” are estimated to be approximately 10 million. Onset of diabetes causes hyperglycemia and also leads to systemic effects, such as high blood pressure and lipid metabolism disorders. Increased healthcare expenditure due to the adverse effects of complications (e.g., diabetic nephropathy, neuropathy, and retinopathy) is a problem. Above all, worsened diabetic nephropathy requires dialysis treatment, which substantially increases the cost of treatment. The monthly healthcare expenditure for dialysis treatment per patient is approximately 400,000 JPY (3607 USD) and approximately 300,000 to 500,000 JPY (2705 USD to 4510 USD) for outpatients undergoing hemodialysis and peritoneal dialysis. This is considered to be one of the factors burdening healthcare expenditures. In 2016, the number of patients who had previously initiated dialysis was 37,252. The most common underlying disease was diabetic nephropathy, and it amounted to 43.2% [6]. Type 2 diabetes is caused by lifestyle. There is an association between body mass index (BMI) as an obese index and type 2 diabetes. The risk of developing diabetes is increased by an elevated BMI. It has also been found that persons who use tobacco products (smokers) are more prone to developing Type 2 diabetes compared to persons who do not use tobacco (non-smokers) [7]. In addition, the number of patients with Type 2 diabetes is higher in people aged 60 to 79 years. Poor exercise habits are associated with age and increase the number of Type 2 diabetic patients. Lifestyle improvements, such as a change in diet and making exercise a habit is necessary to prevent and/or treat Type 2 diabetes. To prevent lifestyle-related diseases, the Ministry of Health, Labour, and Welfare recommends undergoing specific health checkups once a year and advises people who need to improve their lifestyle to receive specific health guidance. However, in 2015, the national implementation rate was 50.1% and 17.5% for specific health examinations and health guidance, respectively. This indicates that effective measures have not been sufficiently performed.

For diabetic patients, hospital visits and lifestyle habit improvement after the onset of diabetes are very important. Unless the issue of lifestyle changes after diagnosis is addressed, the disease may become more severe and cause expensive healthcare expenditures. Therefore, the rationalization of healthcare expenditures in Type 2 diabetic patients is an important problem. In optimizing medical expenses, consideration is required from various factors, such as age and regional characteristics. System dynamics are also used in analysis of future prediction of medical resources [8]. On the other hand, it is possible to use the Bayesian network to quantitatively express the probability of occurrence of a causal relationship involving a complicated route. In addition, compared to neural networks, the calculation process is clear and verification is possible [9]. Typical examples of the use of the Bayesian network are found in the marketing field (e.g., purchasing forecast based on the results of a questionnaire about consumer’s awareness, life, and behavior). There are also many cases where this has been applied to the medical field (e.g., modeling of risk assessment for the level of health guidance by analyzing specific health examination data or a study on the modeling of lifestyle factors which affect bone density) [10,11,12,13]. This study aims to prepare some basic data for the rationalization of healthcare expenditures and determine the characteristics of Type 2 diabetic patients who are more likely to incur high-cost medical expenses.

## 2. Methods

### 2.1. Data Sources

The data of Type 2 diabetic patients (1171 men, 996 women; 2167 patients in total) were extracted from receipt data between April 2016 and March 2017 and the five-year specific health examination data from April 2011 to March 2016, based on the National Health Insurance data obtained from the Iwamizawa City Government in Hokkaido. We extracted the following elements from the data: age, gender, area of residence, number of days provided medical services in a year, number of diseases listed on medical insurance receipts, number of medical examinations undergone in five years, annual healthcare expenditures, and the presence or absence of hospitalization. The reasons for choosing these items are described in the next section.

We chose to look at the number of medical examinations undergone from the viewpoint of the need for the improvement of lifestyle for prevention and treatment of Type 2 diabetes. The number of diseases listed on medical insurance receipts could indicate the presence or absence of diabetes-related complications, such as cerebrovascular disorder and cardiovascular disorder that, in turn, affect the total healthcare expenditures for Type 2 diabetes [14].

### 2.2. Data Analysis

We transformed the subject data to discrete data and classified it into several groups, considered as the observed value distribution. Based on the administrative division of the city, we divided areas of residence into two areas—the urban area and the suburban area in the city. The number of categories for each parameter was set to the equal interval method. The number of days provided medical services was six and the number of diseases was seven. The number of days provided medical services included outpatients and inpatients.

The subject data were modeled by the Bayesian network. The software we used was Bayolink version 7.0.1 (NTT DATA Mathematical Systems Inc., Tokyo, Japan). Greedy Search was used as an algorithm for structural learning that constructs a Bayesian network model from the training data. Akaike’s Information Criterion (AIC) was adopted as the evaluation standard for model building. The search was terminated when the value of the cross-tabulation table (CTT) became 0.01 or less during structural learning. The conditional probability was calculated in this procedure. After constructing the model, the observed value of one parameter was set as the evidence. Probabilistic inference was performed to estimate the conditional probabilities of the other parameters. From the results, we clarified the factors that affected the medical expenses, and verified how much the high-cost medical expenses changed by changing the values of those parameters. We modeled the series of flow (before payment of healthcare expenditure) as follows:

(1) Decision to undergo specific health checkups varies depending on age, gender, and area of residence;
(2) Disease detection, hospital visits, and hospitalization changes based on specific health checkups;
(3) Healthcare expenditure varies depending on the degree of the disease.

Based on this series of flow, we assigned “Attribute information” for the age, gender, and area of residence, “Health/Mibyo (the state without the disease)” for the number of specific healthcare checkups undergone, and “Outpatient and inpatient information” to record the presence or absence of hospitalization, the number of days provided medical services, and the number of diseases listed on medical insurance receipts. These were connected sequentially by arrows, which pointed to the applicable item of healthcare expenditure. In the prepared models, the observed values (i.e., the number of medical examinations undergone; the presence or absence of hospitalization; the number of diseases listed on medical insurance receipts; the number of days provided medical services) were changed and the degree of the changes in the conditional probabilities were analyzed. In this analysis, patients who paid at least 400,000 JPY (3607 USD) a year for medical expenses were considered as patients with high-cost medical expenses. This analysis focused on the changes in the percentage of patients with high-cost medical expenses. The criterion of 400,000 JPY (3607 USD) a year or more was defined as a standard amount, based on the per capita healthcare expenditures of 332,000 JPY (2994 USD).

### 2.3. Ethical Considerations

This study was approved by the Ethics Review Committee of Faculty of Health Sciences, Hokkaido University (17–118). The data used are in an anonymized state that cannot be linked, using data that excludes personally identifiable information.

## 3. Results

**Figure 1:**
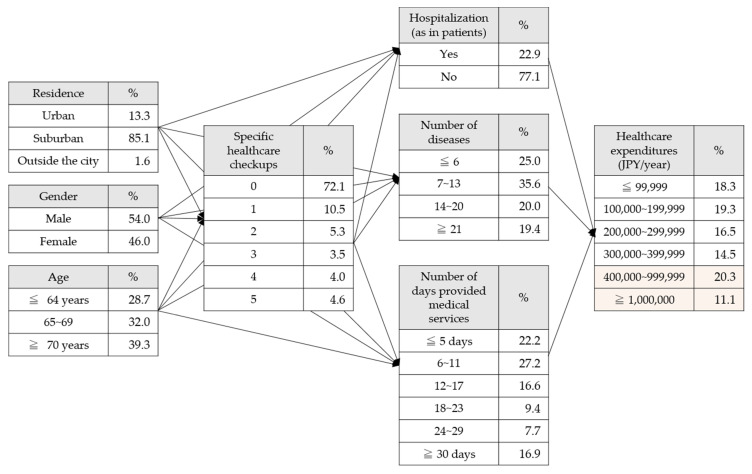
Bayesian network model and the observed values from the data set.

Figure 1 shows the prepared models. The values in Figure 1 are the observed values from the data set.

**Figure 2:**
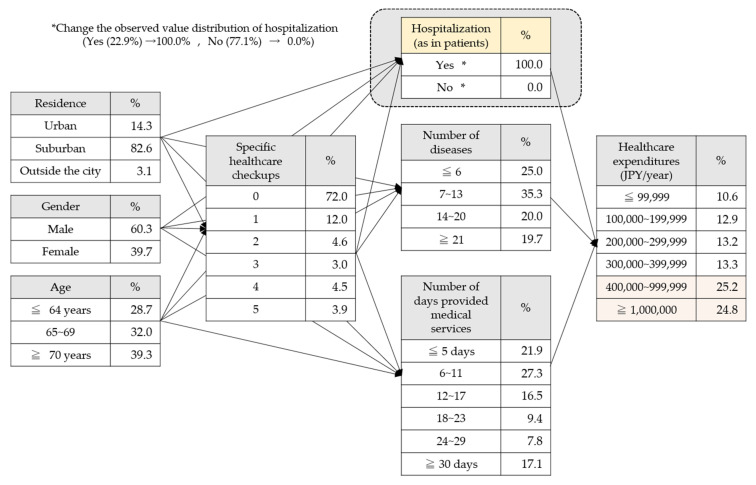
Hypothetical model in which everyone was hospitalized.

The observed values were changed using this model, and the changes in the conditional probabilities of healthcare expenditures were analyzed (Figure 2).

**Figure 3:**
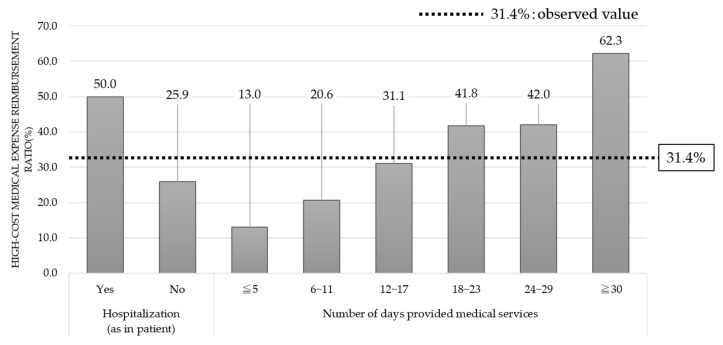
High-cost medical expense ratio (hospitalization and number of days provided medical services).

**Figure 4:**
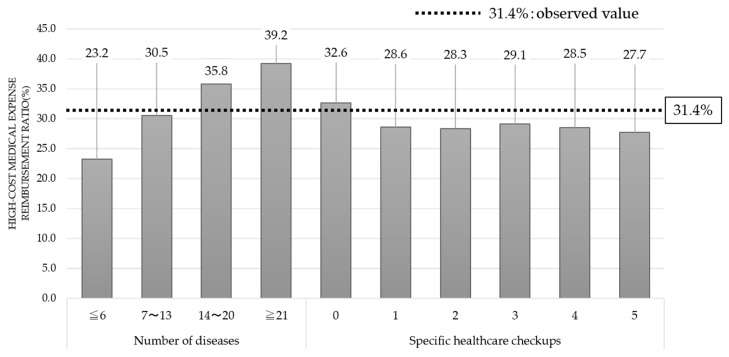
High-cost medical expense ratio (the number of diseases and specific healthcare checkups).

Figure 3 shows the presence or absence of hospitalization; when we hypothesized that all the patients might have been “hospitalized”, the observed values changed. The element for the presence or absence of hospitalization influenced healthcare expenditures. The conditional probabilities of healthcare expenditures were changed by the hypothesis that all the patients had been “hospitalized”. Meanwhile, there was not a significant change in the data of the elements affecting the presence or absence of hospitalization, as compared with healthcare expenditures. By this adjustment, the percentage of high-cost medical expenses for the observed value was 31.4%, while conditional probability changed to 50.0%, which indicated that the observed value exceeded the conditional probability. The same adjustment hypothesizing that 100% of the patients had been “hospitalized” was introduced to the number of days provided medical services, the number of diseases listed on medical insurance receipts, and the number of medical examinations undergone, as well as the presence or absence of hospitalization, and the changes in the percentage of high-cost medical expenses by element is shown in Figure 3 and Figure 4.

Figure 3 shows the change in medical expenses when changing the presence or absence (Yes or No) of hospitalization and the change in medical expenses when changing the number of annual visits. By hypothesizing that all the patients might have been hospitalized, the observed value for the percentage of high-cost medical expenses was 31.4%, while the percentage of high-cost medical expenses increased to 50.0%. In contrast, the percentage of high-cost medical expenses decreased to 25.9% by hypothesizing that all the patients might not have been hospitalized. This demonstrated the possibility that healthcare expenditure for inpatients might be two times more than for patients who had not been hospitalized. The percentage of high-cost medical expenses exceeded 31.4% of the observed value when the number of days provided medical services was hypothesized as follows: patients are provided medical services for 18–23 days a year; patients are provided medical services for 24–29 days a year; and patients are provided medical services for at least 30 days a year. As shown in the graph, a positive relationship was found between the number of days provided medical services in a year and the percentage of high-cost medical expenses. A higher number of days provided medical services led to a higher probability for high-cost medical expenses. When we hypothesized that “all patients had been provided medical services for at least 30 days a year”, the percentage of high-cost medical expenses greatly exceeded the observed value. Conversely, when we hypothesized that all patients “had been provided medical services for five days or less” or all patients “had been provided medical services for 6–11 days”, the percentage of high-cost medical expenses was significantly below the observed value.

Figure 4 shows the changes in healthcare expenditures when changing the number of diseases listed on medical insurance receipts and changes in healthcare expenditures when changing the number of specific healthcare checkups.

When it was hypothesized that all patients had 14–20 diseases listed on medical insurance receipts or all patients had at least 21 diseases listed on medical insurance receipts, the percentage of high-cost medical expenses greatly exceeded the observed value. In addition, there was a positive correlation between a large number of diseases listed on medical insurance receipts and an elevated percentage for high-cost medical expenses. These results suggest that patients who had a higher number of diseases required more treatment and provided medical services, resulting in an increase in healthcare expenditure.

Only when we hypothesized that all patients “had only missed one medical examination” did the percentage of high-cost medical expenses exceed the observed value. On the other hand, for patients who had received at least one medical examination in five years, the percentage of high-cost medical expenses was below the observed value. Based on these results, we can establish that undergoing specific health checkups may reduce expensive healthcare expenditures.

## 4. Discussion

### 4.1. Presence or Absence of Hospitalization

With reference to the fact that the conditional probability of the percentage of high-cost medical expense exceeded the observed value through application of the hypothesis that patients might have been hospitalized, we established that treatment has two purposes for patients who are hospitalized for diabetes: testing and treatment. When complications are suspected, especially in patients with a high blood glucose level, HbA1c, and blood pressure, and who are obese with suspected arteriosclerosis, the number of patients who are hospitalized for examination are usually high. In such cases, the function of the organs in the body is examined and their lifestyle after discharge reviewed. Although the purpose of hospitalization is for tests, hospitalization is sometimes required to educate patients on how to prevent diabetes from worsening. Many of the patients hospitalized for examination show mild symptoms, but patients who are hospitalized for treatment have many serious symptoms, such as poor blood glucose control, symptoms accompanied by dehydration, disturbance of consciousness, and shocks.

Therefore, patients hospitalized for treatment with advanced diabetes and complications are patients that incur expensive healthcare expenditures. Based on the above, hospitalization for treatment increases healthcare expenditure. In this analysis, we did not know whether patients were hospitalized for testing or treatment.

However, by having hypothesized that all patients had been hospitalized, it is thought to be proper that the conditional probability of the percentage of high-cost medical expenses exceeded the observed value.

### 4.2. Number of Days Provided Medical Services

When the number of days provided medical services per year was at least 18, the conditional probability of the percentage of high-cost medical expenses exceeded the observed value. In contrast, since the conditional probability resulting in high-cost medical expenses was less than the observed value when the number of days provided medical services a year was 12–17 or less, healthcare expenditure can be reduced when the number of days provided medical services are around once a month.

In comparison with other countries, it was estimated that the medical cost per outpatient doctor’s consultation in Japan was 86 USD (median was 139.5 USD), which was cheap compared to other countries [15]. On the other hand, the number of annual doctor’s consultations in Japan was 12.8 (median was 6.45), which was reported to be second amongst OECD member countries [5]. This result suggests that the pace of provided medical services exceeding the average in Japan leads to higher medical costs.

### 4.3. Number of Diseases Listed on Medical Insurance Receipts

Regarding the results that the conditional probability of the percentage of high-cost medical expenses exceeds the observed value when the number of diseases listed on medical insurance receipts is at least 14, the results that conditional probability of the percentage of high-cost medical expenses rises with a higher number of diseases listed on medical insurance receipts may be reasonable, because healthcare expenditure is increased by a higher number of complications. There are many reports on comorbidities, healthcare expenditures, and financial burden. Many studies have also been performed in the fields of obesity, chronic obstructive pulmonary disease (COPD), eating disorder-related mental disorders, and so forth [16,17,18]. However, there is no report on the association between the number of diseases and related healthcare expenditure for complications and comorbidities. Thus, we believe the results of our study are unique and important. It is also necessary to consider not only the number of diseases, but also regionality and the types of comorbidities. According to a study by Akena et al., depression is common in diabetic patients in Uganda, which is associated with many harmful outcomes [19]. Additional surveys and analyses are necessary to consider the types of comorbidities (e.g., mental disorders, such as depression) and how they impact healthcare expenditures.

### 4.4. Number of Specific Healthcare Checkups

There are many patients whose Type 2 diabetes progresses slowly without prominent symptoms early in the process. In such cases, the symptoms have already become severe once the disease is detected. In order to notice the onset of Type 2 diabetes, it is necessary to recognize one’s own health condition by medical examinations, such as specific health checkups. Even if it has developed, it is possible to prevent its severity and prevent the onset of complications by carrying out appropriate treatment.

From the viewpoint that early detection can prevent future complications, it is reasonable that only people who have not undergone any specific health examinations in five years may exceed the observed value of the percentage of high-cost medical expenses. Takeuchi reported that healthcare expenditures for elderly outpatients and inpatients among the medical examinees were lower than those among the non-examinees [20]. However, the difference in the number of medical examinations undergone (one or more) did not significantly influence expensive healthcare expenditures. Therefore, it is necessary to analyze the cost-effectiveness of annual specific health examinations.

### 4.5. Limitations

We conducted our survey in only one local government area in Japan. However, the economic cost for patients with Type 2 diabetes is affected by the state of the national economy or the mean age in the country [21]. Consequently, it is necessary to compare our data with data obtained from other local governments in Japan and from other countries.

The fact that it is not possible to consider the influence of the number of days provided medical services on specific visit numbers of checkups is also one of the limitations of the model constructed in this analysis. Patients regularly visiting hospitals often receive similar inspections as specific health checkups at the hospital. In the case of such a patient, it is expected that there is a tendency not to undergo specific health checkups. We need to investigate the proportion of such patients. Furthermore, we think that it is necessary to analyze by incorporating information such as visit status and past medical history into the model used in this study.

## 5. Conclusions

Using a Bayesian network model, the characteristics of Type 2 diabetic patients who are more likely to cause high-cost medical expenses were modeled, analyzed, and extracted. Based on our analysis, the percentage of patients with high-cost medical expenses increased in the following conditions: when the patient “had been hospitalized”, “had been provided medical services at least 18 days a year”, “had at least four diseases listed on medical insurance receipts”, or “had not had a specific health examination in five years”. The increase in the number of days provided medical services had an effect on the high-cost medical expenses, but the effect on the increase in the high-cost medical expenses was greater when hospitalized. It was clarified that proper outpatient treatment after the onset of illness is effective in controlling medical expenses. It was suggested that expensive healthcare expenditures in Type 2 diabetic patients may be reduced if there are national and local government-established measures for patients with such characteristics.
